# Preliminary Evidence of Increased Hippocampal Myelin Content in Veterans with Posttraumatic Stress Disorder

**DOI:** 10.3389/fnbeh.2015.00333

**Published:** 2015-12-02

**Authors:** Linda L. Chao, Duygu Tosun, Steven H. Woodward, Daniela Kaufer, Thomas C. Neylan

**Affiliations:** ^1^Center for Imaging of Neurodegenerative Diseases, Veterans Affairs Medical CenterSan Francisco, CA, USA; ^2^Department of Radiology and Biomedical Imaging, University of CaliforniaSan Francisco, CA, USA; ^3^Department of Psychiatry, University of CaliforniaSan Francisco, CA, USA; ^4^Dissemination and Training Division, National Center for PTSD, VA Palo Alto Health Care SystemCA, USA; ^5^Helen Wills Neuroscience Institute, University of CaliforniaBerkeley, CA, USA; ^6^Department of Integrative Biology, University of CaliforniaBerkeley, CA, USA; ^7^Canadian Institute for Advanced Research (CIFAR)Toronto, ON, Canada; ^8^Mental Health Services, Veterans Affairs Medical CenterSan Francisco, CA, USA

**Keywords:** myelin, hippocampus, post-traumatic stress disorder, imaging, plasticity

## Abstract

Recent findings suggest the formation of myelin in the central nervous system by oligodendrocytes is a continuous process that can be modified with experience. For example, a recent study showed that immobilization stress increased oligodendrogensis in the dentate gyrus of adult rat hippocampus. Because changes in myelination represents an adaptive form of brain plasticity that has a greater reach in the adult brain than other forms of plasticity (e.g., neurogenesis), the objective of this “proof of concept” study was to examine whether there are differences in myelination in the hippocampi of humans with and without post-traumatic stress disorder (PTSD). We used the ratio of T1-weighted/T2-weighted magnetic resonance image (MRI) intensity to estimate the degree of hippocampal myelination in 19 male veterans with PTSD and 19 matched trauma-exposed male veterans without PTSD (mean age: 43 ± 12 years). We found that veterans with PTSD had significantly more hippocampal myelin than trauma-exposed controls. There was also found a positive correlation between estimates of hippocampal myelination and PTSD and depressive symptom severity. To our knowledge, this is the first study to examine hippocampal myelination in humans with PTSD. These results provide preliminary evidence for stress-induced hippocampal myelin formation as a potential mechanism underlying the brain abnormalities associated with vulnerability to stress.

## Introduction

It is well known that our brains are constantly remodeling neuronal pathways as we learn and experience the world around us. Exciting recent findings suggest that this remodeling process is not just a property of neurons, but that oligodendrocytes, the main myelin forming cells in the central nervous system, and their precursors are also adapting and changing with experience (Young et al., 2013; Gibson et al., 2014; Long and Corfas, 2014). Furthermore,there is evidence that the formation of myelin is a continuous and modifiable process that contributes to psychiatric disorders and other diseases that affect cognition (Fields, 2008). For example, a study that examined genes in prefrontal cortex of schizophrenic brains found 35 of 89 abnormally regulated genes were involved in myelination (Hakak et al., 2001).

Alterations in white matter tracts and myelin formation have been noted in numerous mental disorders, including posttraumatic stress disorder (PTSD; e.g., Daniels et al., 2013). There are also hints that stress may affect white matter through the regulation of oligodendrogenesis. For example, glucocorticoid stress hormones are potent inducers of pro-oligodendrogenic transcription factors and increase oligodendrogenesis (Almazan et al., 1986; Barres et al., 1994; Mann et al., 2008) and myelination (Kumar et al., 1989; Tsuneishi et al., 1991; Masters et al., 1994; Zhu et al., 1994; Cheng and de Vellis, 2000; Desarnaud et al., 2000) in oligodendrocyte precursor cell culture. Glucocorticoids have also been shown to dysregulate myelination *in utero* (Antonow-Schlorke et al., 2009). Recently, Chetty et al. (2014) reported that immobilization stress increased oligodendrogensis in the dentate gyrus of adult rat hippocampus. Because changes in myelination represents a form of adaptive brain plasticity that has a far greater reach in the adult brain (Young et al., 2013; Gibson et al., 2014; Long and Corfas, 2014) than other forms of plasticity (e.g., adult neurogenesis), the present study sought to examine whether vulnerability to post-traumatic stress is similarly associated with myelin dysregulation in humans.

Although myelinated axons are located predominately in white matter, they also exist in gray matter (Hildebrand et al., 1993; Nieuwenhuys, 2013). Because myelin co-varies with T1-weighted (T1w) and T2-weighted (T2w) image intensity from magnetic resonance images (MRI) in opposite directions (Yoshiura et al., 2000), this makes it possible to estimate the degree of myelination in the human brain *in vivo* using ratio of T1w/T2w image intensity Glasser and Van Essen (2011). In a validation of this method, Bock et al. (2009); Bock et al. (2011) compared T1 and T1w/T2w image intensity maps to myelin stains in the marmoset monkey. They found that the myelin features of various cortical areas (e.g., the primary visual, auditory, somatosensory, and motor cortices) identified non-invasively with T1w/T2w image intensity corresponded well with myelin stained sections in the same animals. Another validation study showed that a post-mortem T1w/T2w map of human somatosensory cortex had an intensity border closely aligned with the myeloarchitectonically and cytoarchitectonically defined border between areas 4 and 3a as seen in histological stains of the same piece of tissue (Geyer et al., 2011).

The objective of this “proof of concept” study was to use the ratio of T1w/T2w image intensity to examine whether there are differences in the degree of hippocampal myelination in individuals with and without PTSD. Based on the findings of Chetty et al. (2014); who focused on the hippocampus because of its role in regulating memory and emotion and because it is the site of some of the most significant neurological impact of trauma (Sherin and Nemeroff, 2011), we hypothesized that veterans with PTSD would have greater hippocampal myelination than veterans without PTSD.

## Materials and Methods

### Participants

Magnetic resonance imaging data from 38 males veterans (mean age: 43 ± 12 years) were examined for this study. All participants provided written informed consent approved by the University of California at San Francisco and the Veterans Administrations Committees on Human Research.

Twenty veterans included in the current analysis (8 PTSD+, 12 PTSD−) were recruited as part of a larger study investigating the effects of service in the Persian Gulf War on brain structure and brain function. Eighteen other veterans (11 PTSD+, 7 PTSD−) were recruited from the outpatient mental health clinic of the San Francisco Veterans Affairs Medical Center and by advertising in the community. All veterans participating in the study were evaluated by a Ph.D. level clinical interviewer using the Structured Clinical Interview for *DSM-IV* Diagnosis (SCID, First et al., 1995) the Clinician Administered PTSD Scale (CAPS, Blake et al., 1995) and an interview version of the Life Stressor Checklist-Revised (Wolfe et al., 1996) to determine exposure to traumatic events. The Life Stressor Checklist-Revised assesses 21 stressful life events (e.g., experiencing or witnessing serious accidents, illnesses, sudden death, and physical and sexual assault). The SCID was used to diagnose current major depressive disorder (MDD) and to rule out individuals with a lifetime history of psychotic or bipolar disorders and alcohol abuse or dependance within the previous 12 months and drug abuse or dependance within the previous six months. Other exclusion criteria were neurological illness, head trauma with loss of consciousness greater than 10 min, medical disorders affecting brain function, and conditions ineligible for MRI.

All subjects with PTSD had traumatic exposure related to combat. The trauma histories of the subjects without PTSD included 16 individuals who served in combat, two who experienced traumatic accidents, and one who experienced a traumatic physical assault. The groups included a mixture of Gulf War (82%), Iraq (32%), Afghanistan (4%), Beirut (4%), Vietnam (4%), and multiple (7%) theater exposure. Three subjects with PTSD were taking antidepressant medications, two subjects (1 PTSD+, 1 PTSD−) were on antiepileptic medication for neuropathic pain, while four subjects (2 PTSD+, 2PTSD−) had been prescribed atypical antipsychotic medication (i.e., Ziprasidone) for anxiety and depression. Eleven subjects (9 PTSD+, 2 PTSD−) had a diagnosis of current major depressive disorder. Twelve subjects (7 PTSD+, 5 PTSD−) reported a history of alcohol abuse or dependance. Five subjects (2 PTSD+, 3 PTSD−) reported a history of drug abuse or dependance. On average, the episodes of alcohol/drug abuse/dependance occurred 17.6 years (range, 1–42 years) before the study. None of the episodes occurred within a year of the study. Eighteen subjects (8 PTSD+, 10 PTSD−) had Gulf War Illness (GWI) according to the Centers for Disease Control and Prevention (CDC) case definition (Fukuda et al., 1998). Six subjects (3 PTSD+, 3 PTSD−) had suspected exposure to low-levels of sarin as determined by information obtained from the Directorate for Deployment Health Support of the Special Assistant to the Under Secretary of Defense (Personnel and Readiness) for Gulf War Illness Medical Readiness and Military Deployments. US demolition operations at the Khamisiyah ammunition point (case narrative) (2002). The demographics and clinical characteristics of the participants are summarized in Table 1.

**Table 1:** **Sample characteristics**.

	PTSD−	PTSD+
N	19	19
Age (in years)	43.0 (13.8)	42.7 (10.7)
No. (%) Caucasian	10 (53%)	12 (63%)
Education (in years)	14.8 (1.8)	13.7 (1.7)
CAPS	10.3 (9.5)	59.4 (15.8)^**^
HAM-D	5.0 (6.1)	12.3 (5.5)^**^
No. (%) with current MDD	2 (11%)	9 (47%)^*^
No. (%) on psychotropic medication	3 (16%)	6 (32%)
No. (%) with history of alcohol abuse/dependance	5 (26%)	7 (37%)
No. (%) with history of drug abuse/dependance	3 (16%)	2 (11%)
No. (%) Gulf War veterans	13 (68%)	11 (58%)
No. (%) with Gulf War Illness^a^	10 (53%)	8 (42%)
No. (%) with suspected sarin exposure^b^	3 (16%)	3 (16%)

Means (SD) or number (%) reported. Abbreviations: PTSD, post-traumatic stress disorder, CAPS, Clinician Administered PTSD Scale, MDD, major depressive disorder, HAM-D, Hamilton Depression Scale. Significantly different from PTSD− at ^*^p < 0.05, ^**^p < 0.001. ^a^according to Centers for Disease Control and Prevention case definition (Fukuda et al., 1998). ^b^according to the Directorate for Deployment Health Support of the Special Assistant to the Under Secretary of Defense (Personnel and Readiness) for Gulf War Illness Medical Readiness and Military Deployments. US demolition operations at the Khamisiyah ammunition point (case narrative) (2002).

### Measures

We used the total CAPS score to assess current PTSD symptom severity, the Hamilton Depression rating scale (HAM-D; Hamilton, 1967) to assess depressive symptomology.

### MRI Acquisition and Data Processing

Magnetic resonance imaging scans were acquired at the Center for Imaging of Neurodegenerative Diseases at the San Francisco Veterans Affairs Medical Center using a Bruker/Siemens Med-Spec 4T MRI system (Bruker BioSpin, Ettlingen, Germany) equipped with an 8-channel array receiver coil. The MRI scan protocol consisted of a volumetric T1-weighted magnetization prepared gradient echo sequence (repetition time, 2300 ms; time following inversion pulse, 950 ms; echo time, 4 ms; 7° excitation pulses; 1 mm isotropic resolution) and a volumetric T2-weighted turbospin echo sequence (repetition time, 3400 ms; echo time, 403 ms; 109 echoes per k-space segment with variable flip angles; Slice Turbo factor = 2, FOV = 256 mm × 224 mm × 176 mm, 1 mm isotropic resolution, and 176 continuous sagittal slices), acquired during the same scanning session.

The T2w images were registered to the T1w images using FSL’s FLIRT (Jenkinson et al., 2002) with six parameters (rigid body) and the mutual information cost function. Next, the T2w images were resampled using the trilinear interpolation onto T1 imaging space. Finally, the T1w images were divided by the aligned T2w images to estimate myelin content. As proposed by Glasser and Van Essen (2011); T1w and T2w images were not preprocessed for the signal intensity bias related to the sensitivity profile of the radio frequency receiver coils before generating the ratio image. Assuming that the signal intensity bias was the same in both images, the signal intensity bias was mathematically canceled by the ratio operator.

**Figure 1:**
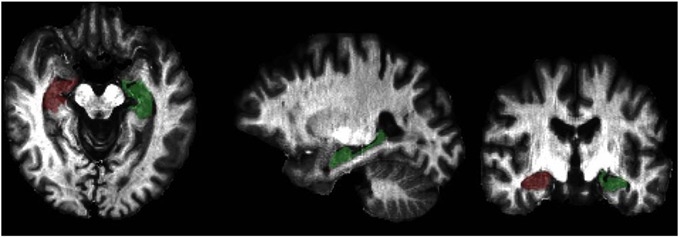
**Graphical representation of the Freesurfer hippocampal region of interest (ROI) overlaid on a T1w/T2w intensity map**.

Because we were interested in estimating the degree of myelination in the hippocampus, we did not perform surface-based analyses of the T1w /T2w data as Glasser and Van Essen (2011) had done. Instead, we conducted a region of interest (ROI) analysis using the Freesurfer version 4.5 tissue boundary delineation of the hippocampus (Fischl et al., 2004). Specifically, we extracted T1w/T2w values from the T1w/T2w images, which were in T1 space, from the Freesurfer hippocampal ROI (see Figure 1). Because we did not have *a priori* hypotheses about laterality differences and because Chetty et al. (2014) did not report laterality differences, we averaged the estimates of left and right hippocampal myelin to reduce the number of measurements.

### Analyses

Statistical analyses of the demographic, clinical, and T1w/T2w values were performed using IBM SPSS Statistics, version 23. Demographic, descriptive, and clinical characteristics were compared across the groups with student’s *t*-test for continuous variables and Fisher’s exact of independance for categorical variables. Because the Shaprio–Wilks test indicated that the variables of interest (hippocampal T1w/T2w ratio and CAPS) were not normally distributed, we used the nonparametric Mann-Whitney test to examine group differences in hippocampal myelin content and Spearman’s Rank-Order correlation to examine the relationship between hippocampal myelin content and current PTSD symptoms severity (i.e., CAPS).

## Results

Table 1 summarizes the demographic and clinical characteristics of the 19 PTSD+ and the 19 PTSD− subjects. As expected, there were no significant group differences in age, years of education, race, history of alcohol and/or drug abuse/dependance, number of GW veterans, number of veterans with Gulf War Illness, or number of veterans with suspected sarin exposure. The PTSD+ group had higher CAPS (*t* = 11.62, *df* = 36, *p* < 0.001) and HAM-D (*t* = 0.83, *df* = 36, *p* < 0.001) scores and a higher incidence of current MDD (*p* = 0.03, Fisher’s exact test).

**Figure 2:**
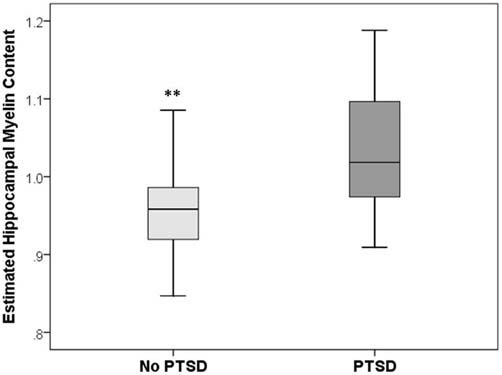
**Box and whisker plots of estimated hippocampal myelin content in PTSD+ (dark gray) and PTSD− (light gray) veterans**. ^**^Group difference significant at *p* < 0.01.

There was a significant group difference in the estimate of hippocampal myelination (*p* = 0.006, Mann-Whitney *U*-test; see Figure 2). Bivariate correlations revealed significant positive correlations between estimates of hippocampal myelination, CAPS (Spearman’s *ρ* = 0.38, *p* = 0.019), and HAM-D (Spearman’s *ρ* = 0.46, *p* = 0.004) scores.

## Discussion

To our knowledge, this is the first study to examine the degree of hippocampal myelination in humans with PTSD. We found that veterans with PTSD had a significantly greater degree of hippocampal myelination compared to matched, trauma-exposed veterans without PTSD. The degree of hippocampal myelination was also significantly and positively correlated with current PTSD symptom severity. These findings, consistent with the recent report in rats by Chetty et al. (2014); suggest that vulnerability to traumatic stress may similarly dysregulate myelination in the human hippocampus.

Chetty et al. (2014) showed that stress stimulated the production of adult oligodendrocytes from neural stem cells in the rat dentate gyrus. It is unclear if a similar mechanism or if other mechanisms are responsible for the increased hippocampal myelin content that we observed in veterans with PTSD in the current study. For example, stress may promote myelin synthesis in pre-existing adult oligodendrocytes (Young et al., 2013). Alternatively, stress may stimulate the activity of oligodendrocyte precursor cells in the parenchyma and the subventricular zone. Whatever the mechanism, it is significant that we found hints of maladaptive myelin development in humans with vulnerability to stress because: (i) maladaptive myelin formation has been implicated in other psychiatric disorders (Fields, 2008); (ii) Changes in myelination represents a form of plasticity that has a greater reach in the brain regions implicated in PTSD (i.e., amygdala, anterior cingulate, insula, and orbitofrontal region (Rauch et al., 2006; Shin et al., 2006) than adult neurogenesis, which is limited to a small population of neuronal stem cells (Schoenfeld and Gould, 2012); and (iii) PTSD has been associated with white matter alterations (Daniels et al., 2013).

Although our results concern myelination in the hippocampus, a gray matter structure, as noted in the introduction, myelinated axons are located predominately in white matter. There are several white matter tracts associated with the hippocampus and medial temporal lobe (MTL): the performant path emerges from the entorhinal cortex and ends in the dentate gyrus in the hippocampus (Knowles, 1992). The fornix contains hippocampal projections to the mammillary bodies and anterior nuclei of the thalamus (Nowrangi and Rosenberg, 2015). The ventral cingulum bundle connects parahippocampal gyrus to the posterior cingulate cortex (Jones et al., 2013). Although it does not extend into the hippocampus, the uncinate fasciculus connects the anterior temporal lobe to lateral orbitofrontal cortex through a direct, monosynaptic, bidirectional pathway (Von Der Heide et al., 2013).

Diffusion tensor imaging (DTI) is an imaging technique that allows for the interrogation of the microstructural integrity of white matter (Le Bihan, 2003). A few DTI studies have attempted to investigate the performant pathway *in vivo* in patients with mild cognitive impairment (MCI), an intermediate stage between normal aging and Alzheimer’s disease (AD; Morris et al., 2001), and AD. However, these studies were limited by low resolution and the inability to specifically identify performant pathway fibers (Kalus et al., 2006; Rogalski et al., 2009). Recently, Yassa et al. (2010) used ultrahigh-resolution microstructural DTI with submillimeter resolution to identify diffusion signals unique to the performant path. However, this technique has yet to be applied to populations with PTSD.

In a meta-analysis of seven whole-brain DTI studies of trauma-exposed adults, Daniels et al. (2013) identified significant decreases and increases in fractional anisotropy (FA), a DTI metric commonly used as a proxy measure for white matter integrity (Alexander et al., 2007), in the dorsal cingulum. The largest cluster in the right dorsal cingulum pertained to FA decreases; however, Daniels et al. (2013) also identified clusters of FA increases and FA decreases bilaterally in other sections of the dorsal cingulum. In a longitudinal study of treatment-related DTI differences in remitted and persistent PTSD patients and combat controls, Kennis et al. (2015) suggested that higher FA in the dorsal cingulum bundle may be an acquired feature of persistent PTSD that develops over time. Kennis et al. (2015) also found significant group by time interactions in the hippocampal (ventral) cingulum, fornix, and stria terminalis, which they suggested might reflect the differential effects that PTSD treatment may have on FA in these white matter tracts in remitted vs. persistent PTSD patients.

It is noteworthy that the Kennis et al. (2015) did not find significant overall effects of group or time in hippocampal/MTL white matter tracts. In fact, no DTI studies of populations affected by PTSD to date have reported significant DTI changes in hippocampal or MTL white matter tracts. In contrast, numerous DTI studies of patients at risk for AD (Mielke et al., 2012; Douaud et al., 2013) and MCI (Fellgiebel and Yakushev, 2011; Liu et al., 2011; Zhuang et al., 2012, 2013; Ito et al., 2015) have reported significant DTI changes in the fornix and hippocampal cingulum. It is well documented that the hallmarks of AD pathology begin in the transentorhinal region of the brain (Braak and Braak, 1991). Therefore, it may be there are greater structural white matter alterations in the hippocampus and MTL of patients with MCI and pre-clinical AD than patients with PTSD. Alternatively, the lack of PTSD−related DTI findings in the hippocampus and MTL may be attributable, as least in part, to the fact that most DTI studies of PTSD have only examined FA.

It has been suggested that other DTI parameters (i.e., axial and radial diffusivity) may be more sensitive to changes in axonal morphology and myelination than FA (Tyszka et al., 2006). For example, there is suggestive evidence that radial diffusivity is particularly sensitive to changes in myelination (Song et al., 2002, 2003; Song et al., 2005). Because few DTI studies of PTSD have considered radial diffusivity, this may, at least in part, account for why past DTI studies of PTSD have not reported significant findings in the hippocampus or MTL. As a case in point, in a previous DTI study of veterans with and without PTSD, 18 of whom are part of the present report, our group did not find any significant medial temporal FA differences (Schuff et al., 2011). However, this study did not examine radial diffusivity. Thus, future studies will need to determine if vulnerability to trauma-related stress and PTSD has a similar effect on myelination in hippocampal white matter tracts as it appears to have in hippocampal gray matter.

In the central nervous system, myelin insulates and protects axons and facilitates the conduction of nerve impulses (Barkovich, 2000). Therefore, the loss of myelin can result in reduced nerve conduction velocity, reduced millisecond axonal precision, and reduced range of cortical synchrony. These, in turn, could impact the overall speed of mental processes, spike-timing dependent plasticity, and/or functional connectivity (Nave and Ehrenreich, 2014). However, having an excess of myelin, particularly in gray matter structures, may also have negative consequences. For example, there is evidence that myelin inhibits synapse formation and reduces plasticity in the central nervous system (Chen et al., 2000; McGee and Strittmatter, 2003; McGee et al., 2005), that oligodendrocytes inhibit axon growth cones (Fawcett et al., 1989; Bandtlow et al., 1990; Morganti et al., 1990), and that oligodendrocytes precursor cells are repulsive for growing axons (Chen et al., 2002a, b). Because the formation and elimination of axons and synapses are critical for learning and memory (Kleim et al., 2002, 2004; Holtmaat and Svoboda, 2009; Xu et al., 2009; Yang et al., 2009; Boele et al., 2013), one consequence of excessive myelin, particularly in the hippocampus, may be a reduced ability to learn and remember (Squire and Alvarez, 1995). In this context, it is noteworthy that memory disturbances are predominant in the presentation of PTSD (Samuelson, 2011) and are part of the diagnostic criteria (American Psychology Association).

The present findings should to be considered within the context of a number of limitations: First, the sample was small and these preliminary findings of increased hippocampal myelination in individuals with PTSD will need to be replicated in a larger sample. Second, the inclusion of only male veterans may limit generalizability of the findings. Third, the Glasser and Van Essen model, which depends on a relatively simplistic model of bound and unbound water, may not be unambiguously specific for myelin. Fourth, we did not perform a surface-based analyses of the T1w/T2w ratio data as described by Glasser and Van Essen (2011) because we were primarily interested in examining myelination in the hippocampus. Thus, future research will need to investigate whether vulnerability to stress promotes myelin changes in other brain regions that have been implicated in PTSD (i.e., amygdala, anterior cingulate, insula, and orbitofrontal cortex; Rauch et al., 2006; Shin et al., 2006). Also, because PTSD has been associated with white matter alterations (Daniels et al., 2013), future investigations should examine/compare myelin changes in gray and white matter using T1w/T2w ratio and radial diffusivity, which has been proposed be sensitive to alterations in myelination (Song et al., 2002, 2003; Song et al., 2005). Fifth, we cannot be certain of the specificity the present findings to PTSD because many of the PTSD subjects had comorbid depression. However, assembling a PTSD group that is free of depressive symptoms is unlikely to generalize. Furthermore, there has been evidence that calls into question whether PTSD and depression are distinct entities among individuals exposed to trauma given the common criterion symptoms (Elhai et al., 2011). Other limitations include potential partial volume effects and the cross-sectional nature of the study, which limits our ability to determine causality and the subjective nature of patient history and clinical scores, which may be biased by under- or over-reporting. These limitations notwithstanding, the current findings, if replicated, suggest that stress-induced myelin formation in the hippocampus may be a potential mechanism underlying the structural and functional brain abnormalities associated with vulnerability to stress. Longitudinal research will be needed to better understand how myelin changes in gray and white matter brain structures throughout the course of PTSD and in response to treatment for PTSD.

## Conflict of Interest Statement

Dr. Neylan has consulted for Genentech and received study medication from Actelion for a trial funded by the Department of Defense and study medication from Glaxo Smith Kline for a trial funded by the Department of Veterans Affairs. The remaining authors declare no conflicts of interest.
